# Identification of risk factors for the progression of age-related macular degeneration: a systematic review and meta-analysis of cohort studies

**DOI:** 10.3389/fmed.2025.1544765

**Published:** 2025-07-23

**Authors:** Moqi Tian, Baike Zhang

**Affiliations:** ^1^The Fifth Clinical Medical College of Henan University of Chinese Medicine, Zhengzhou, China; ^2^Department of Ophthalmology, 988th Hospital of the Joint Logistics Support Force, Zhengzhou, China

**Keywords:** age-related macular degeneration, risk factors, progression, systematic review, meta-analysis

## Abstract

**Background:**

Age-related macular degeneration (AMD) is a retinal degenerative disease that primarily affects the elderly population and is a leading cause of vision loss in older adults. There is a lack of research comprehensively examining the risk factors for AMD progression. This study aimed to identify the risk factors influencing the development of AMD using a meta-analysis approach.

**Methods:**

We systematically searched the PubMed, Embase, and Cochrane Library databases from their inception until November 2024. Summary effect estimates were assigned as odds ratios (ORs) with 95% confidence intervals (CIs) using a random effects model. Further exploratory analyses included sensitivity and sub-group analyses.

**Results:**

Eighteen cohort studies involving 38,697 individuals were included in the final meta-analysis. We noted male versus female was associated with a reduced risk of AMD (OR: 0.84; 95% CI: 0.72–0.98; *p* = 0.027). The identified risk factors for AMD included per 5-year increment in age (OR: 1.14; 95% CI: 1.09–1.19; *p* < 0.001), current smoking (OR: 1.28; 95% CI: 1.07–1.52; *p* = 0.007), alcohol intake (OR: 1.30; 95% CI: 1.00–1.67; *p* = 0.046), per 1 mmol/L increment in high-density lipoprotein (OR: 1.21; 95% CI: 1.08–1.36; *p* = 0.001), total drusen >10% of the grid (OR: 7.85; 95% CI: 4.66–13.23; *p* < 0.001), presence of depigmentation (OR: 6.39; 95% CI: 2.48–16.44; *p* < 0.001), presence of hyperpigmentation (OR: 6.03; 95% CI: 1.94–18.73; *p* = 0.002), and >10 small drusen (OR: 7.21; 95% CI: 2.10–24.72; *p* = 0.002).

**Conclusion:**

This study systematically identified the risk factors for AMD progression, and exploratory analyses were performed to determine the risk factors for early and late AMD. Patients at high risk of AMD should be monitored to improve modifiable risk factors and slow the progression of AMD.

**Systematic review registration:**

INPLASY platform (INPLASY2024120036).

## Introduction

Age-related macular degeneration (AMD) is a degenerative disease of the macula that affects approximately 200 million people globally and is a significant public health issue (1). AMD is a leading cause of severe and irreversible vision loss, accounting for 8.7% of total global blindness, second only to cataracts and uncorrected refractive errors (2, 3). With the increasing global life expectancy, a growing aging population, and declining mortality rates in most countries and regions, the prevalence and burden of AMD are expected to increase, increasing its significance as a public health issue (2). It is predicted that by 2040, the number of people affected by AMD will increase to 288 million (1). Given its irreversible nature and the lack of effective prevention strategies, research on modifiable risk factors and understanding the mechanisms leading to AMD are crucial for promoting health and longevity (4, 5).

AMD is a multifactorial disease (6) characterized by retinal pigment epithelial changes and drusen formation. Clinically, AMD is classified into early (Small drusen and/or retinal pigment epithelium mottling), intermediate (large drusen or retinal pigment epithelium depigmentation/hyperpigmentation), and late AMD (geographic atrophy or neovascularization, leading to retinal fluid accumulation) (7). Late-stage AMD is associated with significant vision loss. The rate of depression in patients with AMD is twice that of the general population, which is further exacerbated by the loss of independence and leisure activities (8). Studies have shown that in addition to genetic factors, age and processes that promote oxidative stress play significant roles in the development and progression of AMD (9, 10). Other risk factors include smoking, obesity, poor dietary habits, and cardiovascular risk factors (2, 11). Although there are currently no effective treatments for dry AMD, neovascular (wet) AMD can be managed with intravitreal injections of anti-vascular endothelial growth factor inhibitors, which can slow the disease progression (12). Therefore, investigating risk factors for the development and progression of AMD is crucial for early diagnosis and delayed disease progression.

Existing meta-analyses focused on incident AMD but lacked systematic evaluation of progression risk factors, particularly for early vs. late stages (13). Given the growing AMD burden, methodological inconsistencies in prior research, and lack of stage-specific risk factor analyses, this systematic review and meta-analysis aims to: (1) comprehensively synthesize evidence on AMD progression risk factors; (2) identify modifiable factors with high clinical impact; and (3) differentiate risk profiles for early vs. late AMD stages.

## Methods

### Data sources, search strategy, and selection criteria

This systematic review and meta-analysis were conducted in accordance with the Preferred Reporting Items for Systematic Reviews and Meta-Analyses statement published in 2020 (14). This study was registered in INPLASY platform (no: INPLASY2024120036). Cohort studies that investigated factors influencing the development and progression of AMD were eligible for inclusion with no restrictions on language or publication status (published, in press, or ongoing). We searched electronic databases, including PubMed, EmBase, and the Cochrane Library, for articles published from inception up to November 2024, using the following search terms: “age-related macular degeneration” and “risk factors” (Supplementary material 1). We manually searched the reference lists of all relevant primary literature and reviewed articles to identify eligible studies. Medical subject headings, methods, patient populations, study designs, exposure factors, and outcome variables were used to identify relevant studies.

The literature search and screening were independently conducted by two authors using standardized methods. The lead author resolved disagreements until a consensus was reached. Studies were included in the analysis if they met the following criteria: (1) cohort study design; (2) participants did not have AMD at the start of the study; (3) number of reported specific risk factors for AMD ≥ 2; and (4) reported adjusted effect sizes [risk ratios (RRs), hazard ratios (HRs), or odds ratios (ORs)] with 95% confidence intervals (CIs). When the same population was reported in multiple studies, the one with the most comprehensive data was selected. Additionally, if a study reported multiple adjusted outcomes, we chose the maximally adjusted effect size for analysis. Cross-sectional and case–control studies were excluded as they cannot establish a causal relationship between risk factors and AMD.

### Data collection and quality assessment

The collected data included the first author’s name, publication year, geographic location, study name, study period, sample size, age, proportion of male participants, AMD definition criteria, follow-up duration, adjusted factors, and effect estimates with their 95% CI. The Newcastle–Ottawa Scale (NOS) is a comprehensive tool used in meta-analyses to assess the quality of observational studies (15). The NOS has three subscales: selection (four items), comparability (one item), and outcome (three items). A “star system” (ranging from 0 to 9) was also developed to evaluate the methodological quality of observational studies. Data extraction and quality assessment were performed independently by two authors. Another author reviewed and adjudicated the information based on original studies.

### Statistical analysis

We assessed the risk factors for the occurrence and development of AMD based on effect estimates (ORs, RRs, or HRs) and the 95% CIs reported in each study. When combining the effect sizes of these risk factors, we used a random-effects model to account for heterogeneity among the different studies (16, 17). To further explore the degree of this heterogeneity, we utilized the *I*^2^ and Q statistics as measures, defining *I*^2^ ≥ 50% or *p* < 0.10 as the threshold for significant heterogeneity (18, 19). Moreover, sensitivity analyses were conducted by sequentially excluding each study from the meta-analysis to verify the robustness of the results (20). We performed subgroup analyses based on the AMD stage for risk factors included in at least five studies. Differences between subgroups were compared using the interaction *p*-value test, assuming the data are normally distributed (21). Additionally, we employed several strategies to detect potential publication bias, including visual inspection of funnel plots and quantitative assessments using Egger’s regression and Begg’s rank correlation tests (22, 23). All statistical tests mentioned above used two-tailed *p*-values, with *p* < 0.05 considered statistically significant. All data analyses were conducted using the STATA software (version 12.0; StataCorp LP, College Station, TX, United States).

## Results

### Literature search

**Figure 1:**
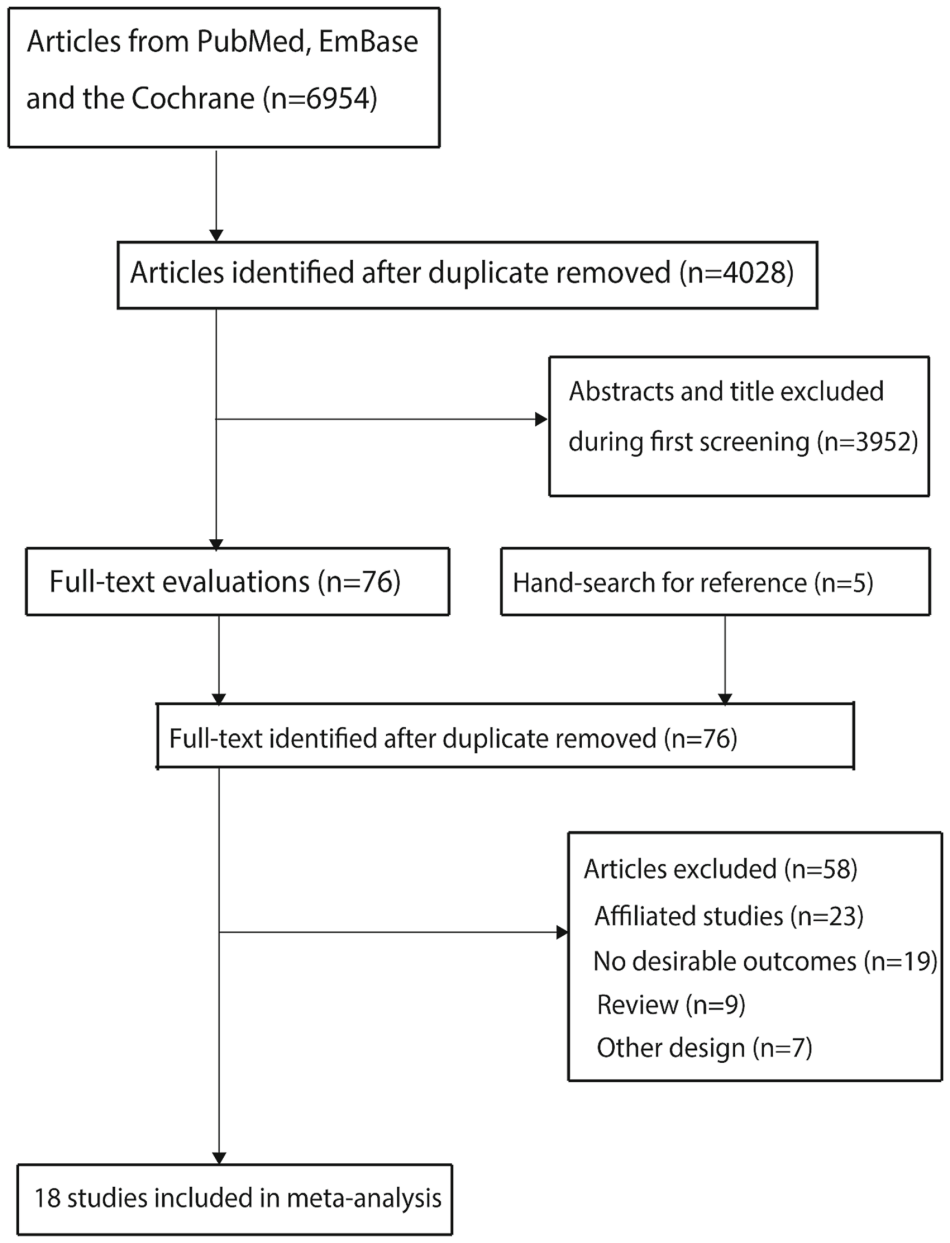
Flow diagram of the literature search and trial selection process.

The results of the study selection process are shown in Figure 1. Our initial electronic search yielded 6,954 articles. After removing duplicates and irrelevant studies, 4,028 articles remained. After screening based on titles and abstracts, 3,952 studies were excluded. We then conducted a full-text assessment of the remaining 76 studies, and 58 were excluded. Consequently, 18 studies were included in the final analysis (24–41). Five potentially relevant articles identified through manual searches were already included among those assessed at the full-text stage and thus were not included. The general characteristics of the included studies and their participants are summarized in Table 1.

**Table 1:** The baseline characteristics of included studies and involved participants.

Study	Region	Study name	Study period	Sample size	Age (years)	Male (%)	AMD grading	Follow-up	Adjusted factors	NOS
Klaver et al. (24)	Europe	Rotterdam Study	1990–1994	4,953	≥55.0	41.5	IGS	2.0 years	Age, baseline stage of AMD, and follow-up duration	8
Delcourt et al. (25)	Europe	PLOA	1995–2000	1,424	≥60.0	NA	IGS	5.0 years	Age	6
Leske et al. (26)	North America	Barbados Eye Study	1987–2003	2,356	40.0–84.0	40.0	Reading Center in Baltimore	9.0 years	Age	7
Klein et al. (27)	North America	BDES	1988–2005	2,119	43.0–86.0	NA	WAMDGS	15.0 years	Age	8
Chang et al. (28)	North America	SEEP	1993–1995	1937	65.0–84.0	41.4	SEEP	2.0 years	Age, sex, and race	7
Yasuda et al. (29)	Asia	Hisayama Study	1998–2007	1,401	≥40.0	37.4	WAMDGS+IGS	9.0 years	Age, gender, smoking habit, and white blood cells	8
Coleman et al. (30)	North America	SOP	1997–2004	1,958	≥65.0	0.0	WAMDGS	5.0 years	Alcohol consumption, current smoking, age, race, hypertension, walking for exercise, and study sites	8
You et al. (31)	Asia	Beijing Eye Study	2001–2006	3,049	≥40.0	43.5	IGS	5.0 years	Age, refractive error, and scleral spur distance	7
Jonasson et al. (32)	Europe	AGES	2002–2011	2,868	≥67.0	42.4	WAMDGS	5.0 years	Age, sex	8
Joachim et al. (33)	Oceania	BMES	1992–2010	2,474	≥49.0	42.4	WAMDGS	15.0 years	Age, sex, smoking, CFH, ARMS2 polymorphisms, and fish consumption	8
Cheung et al. (34)	Asia	SMES	2006–2013	1,809	≥40.0	45.6	WAMDGS	6.0 years	Age, gender, smoking status, hypertension, and diastolic blood pressure	7
Bastawrous et al. (35)	Africa	NEDCS	2007–2013	1,282	≥50.0	50.5	IGS	6.0 years	Age, sex, BMI, location, SES quartile, smoking status, hypertension, DM, alcohol use, ethnic group, educational level	8
Saunier et al. (36)	Europe	ALIENOR	2006–2012	659	≥73.0	37.3	IGS	3.8 years	Age, and sex	7
Foo et al. (37)	Asia	SIES	2007–2015	2,105	≥40.0	49.7	WAMDGS	6.0 years	Age, gender, hypertension, total cholesterol, CVD, BMI, smoking status, alcohol consumption frequency, serum CRP and ARMS2 genetic loci	7
Mao et al. (38)	Asia	THES	2006–2013	5,048	≥30.0	45.1	WAMDGS	6.0 years	Age, sex, blood pressure, hypertension, DM, history of stroke, BMI, TC, HDL, LDL, TG, smoking status, drinking status, refractive error, axial length, and corneal curvature radius	7
Jiang et al. (39)	Asia	CJFH	2015–2020	324	≥60.0	54.0	WAMDGS	10.0 years	Age, sex, DM, hypertension, hyperlipidemia, smoking, DR, BMI, HbA1c, FBG, CHO, TG, HDL, LDL, UA, Cr	6
Brandl et al. (40)	Europe	KORA	1999–2018	1,513	35.0–95.0	51.5	WAMDGS	3.0–18.0 years	Age, sex, smoking status, HDL	6
Ludtke et al. (41)	Europe	SHIP-TREND-1	2008–2019	1,418	28.0–89.0	46.0	WAMDGS	8.0 years	Age	6

### Study characteristics

A total of 38,697 individuals were included in the 18 studies, and the sample sizes ranged from 324 to 5,048. The follow-up durations ranged from 2.0 to 15.0 years. Twelve studies were conducted in Europe, North America, Oceania, and Africa; the remaining six were performed in Asia. Study quality was assessed using the NOS: seven studies had eight stars, seven studies had seven stars, and four studies had six stars.

### Sex, age, and body mass index

**Figure 2:**
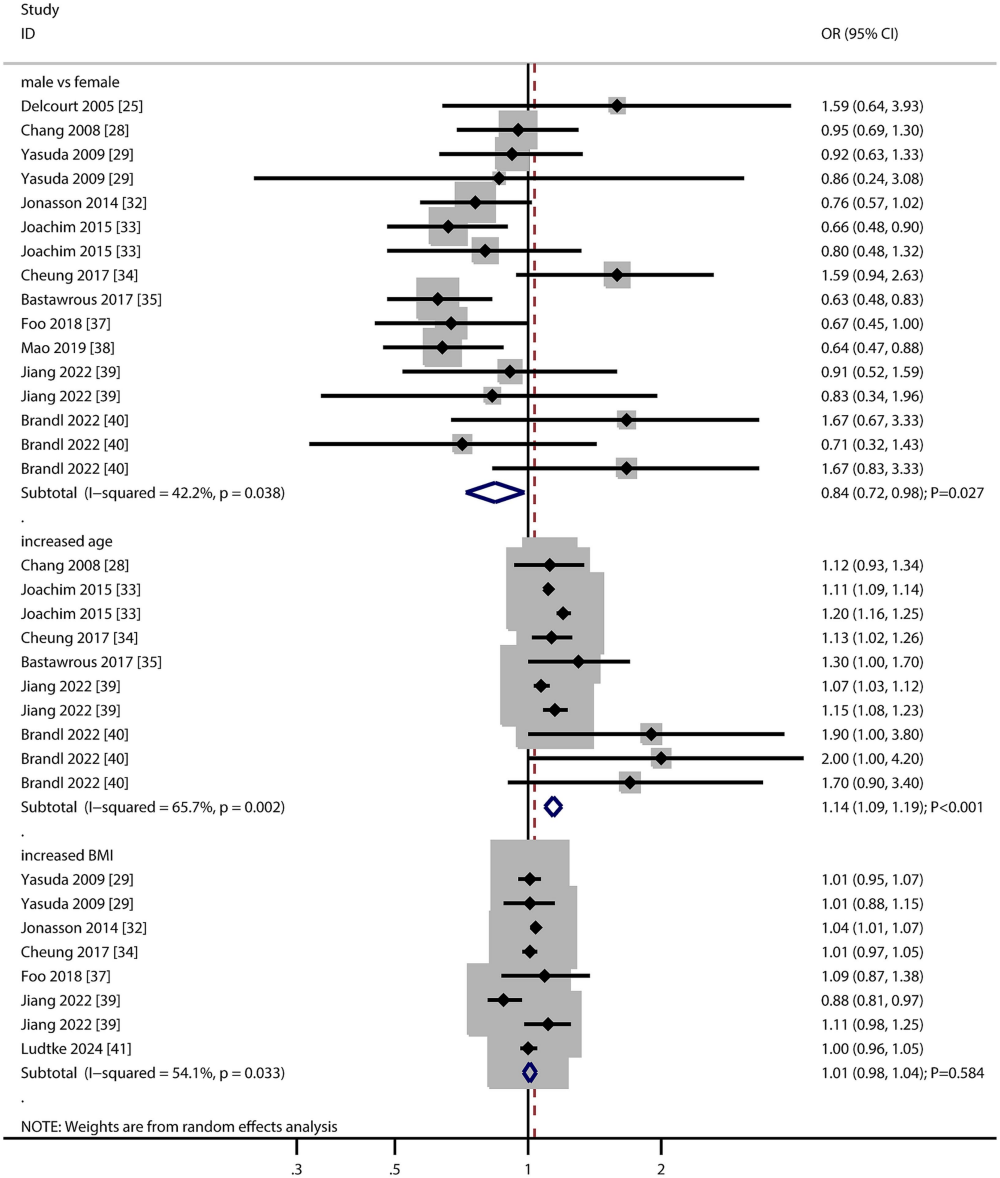
Association of sex, age, and body mass index (BMI) with the risk of age-related macular degeneration (AMD).

The number of studies reporting the association of sex, age, and body mass index (BMI) with AMD risk was 11, six, and six, respectively (Figure 2). We noted that male versus female was associated with a lower risk of AMD (OR: 0.84; 95% CI: 0.72–0.98; *p* = 0.027), and potential heterogeneity was observed (*I*^2^ = 42.2%; *p* = 0.038). The sensitivity analysis indicated that the pooled conclusion was unstable due to the marginal 95% CI (Supplementary material 2). When stratified according to the AMD stage, there was no significant association between sex and the risk of early or late AMD (Table 2). There was a significant publication bias for the association between sex and the risk of AMD (*p* value for Egger: 0.019; *p* value for Begg: 0.034; Supplementary material 3), and a significant association was not observed after adjusting for potential publication bias using the trim and fill method (42).

**Table 2:** Subgroup analyses for risk factors according to AMD stage.

Factors	Subgroup	No of studies	OR and 95%CI	*p* value	*I*^2^ (%)	*p* value for *I*^2^	*p* value between subgroups
Sex (male vs. female)	Early	6	0.96 (0.73–1.25)	0.747	56.8	0.023	0.498
	Late	3	0.81 (0.54–1.23)	0.327	0.0	0.993	
Age (per 5 years increment)	Early	4	1.11 (1.06–1.16)	<0.001	46.5	0.096	0.042
	Late	2	1.18 (1.14–1.23)	<0.001	19.2	0.266	
BMI (per kg/m^2^ increment)	Early	4	0.98 (0.92–1.05)	0.592	64.8	0.036	0.180
	Late	2	1.06 (0.97–1.17)	0.191	4.5	0.306	
Current smoking	Early	9	1.16 (0.98–1.38)	0.077	0.0	0.621	0.001
	Late	5	2.61 (1.66–4.10)	<0.001	0.0	0.410	
Alcohol intake	Early	3	1.38 (1.03–1.85)	0.033	43.4	0.171	0.658
	Late	2	1.13 (0.49–2.60)	0.778	68.8	0.074	
Hypertension	Early	6	0.99 (0.91–1.08)	0.812	12.3	0.336	0.409
	Late	3	1.03 (0.99–1.07)	0.136	0.0	0.659	
DM	Early	5	0.98 (0.82–1.16)	0.781	31.5	0.211	0.492
	Late	3	1.23 (0.66–2.30)	0.511	48.1	0.146	
TC	Early	4	0.99 (0.95–1.03)	0.566	0.0	0.817	0.712
	Late	1	1.00 (0.97–1.04)	1.000	–	–	
HDL	Early	4	1.14 (1.04–1.26)	0.005	0.0	0.459	0.047
	Late	1	1.95 (1.16–3.28)	0.012	–	–	
LDL	Early	3	0.95 (0.85–1.07)	0.421	0.0	0.474	0.006
	Late	1	2.14 (1.21–3.78)	0.009	–	–	
TG	Early	3	0.92 (0.79–1.08)	0.329	61.2	0.076	0.959
	Late	1	0.91 (0.62–1.33)	0.628	–	–	

The summary result indicated per 5-year increment in age was associated with an increased risk of AMD (OR: 1.14; 95% CI: 1.09–1.19; *p* < 0.001), and significant heterogeneity across included studies was observed (*I*^2^ = 65.7%; *p* = 0.002). The sensitivity analysis indicated that the pooled conclusion was stable and not altered by the sequential removal of a single study (Supplementary material 2). The subgroup analysis revealed that the association between 5-year increment in age and the risk of AMD persisted in the early and late AMD groups, and the difference in this association was statistically significant (Table 2). There was no significant publication bias regarding the association per 5-year increment in age (*p* value for Egger: 0.137; *p* value for Begg: 0.074; Supplementary material 3).

There was no significant association per 1 kg/m^2^ increment in BMI with the risk of AMD (OR: 1.01; 95% CI: 0.98–1.04; *p* = 0.584), and significant heterogeneity was seen across included studies (*I*^2^ = 54.1%; *p* = 0.033). The sensitivity analysis revealed that every 1 kg/m^2^ increment in BMI was associated with an increased risk of AMD (Supplementary material 2). The subgroup analysis found that per 1 kg/m^2^ increment in BMI was not associated with the risk of early and late AMD (Table 2). There was no significant publication bias for the association between each 1 kg/m^2^ increment in BMI and the risk of AMD (*p* value for Egger: 0.636; *p* value for Begg: 0.902; Supplementary material 3).

These findings suggest that female sex may be a modest risk factor for AMD, though heterogeneity and publication bias warrant caution. Age remains a robust and consistent risk factor, underscoring the need for age-stratified screening strategies.

### Smoking and alcohol intake

**Figure 3:**
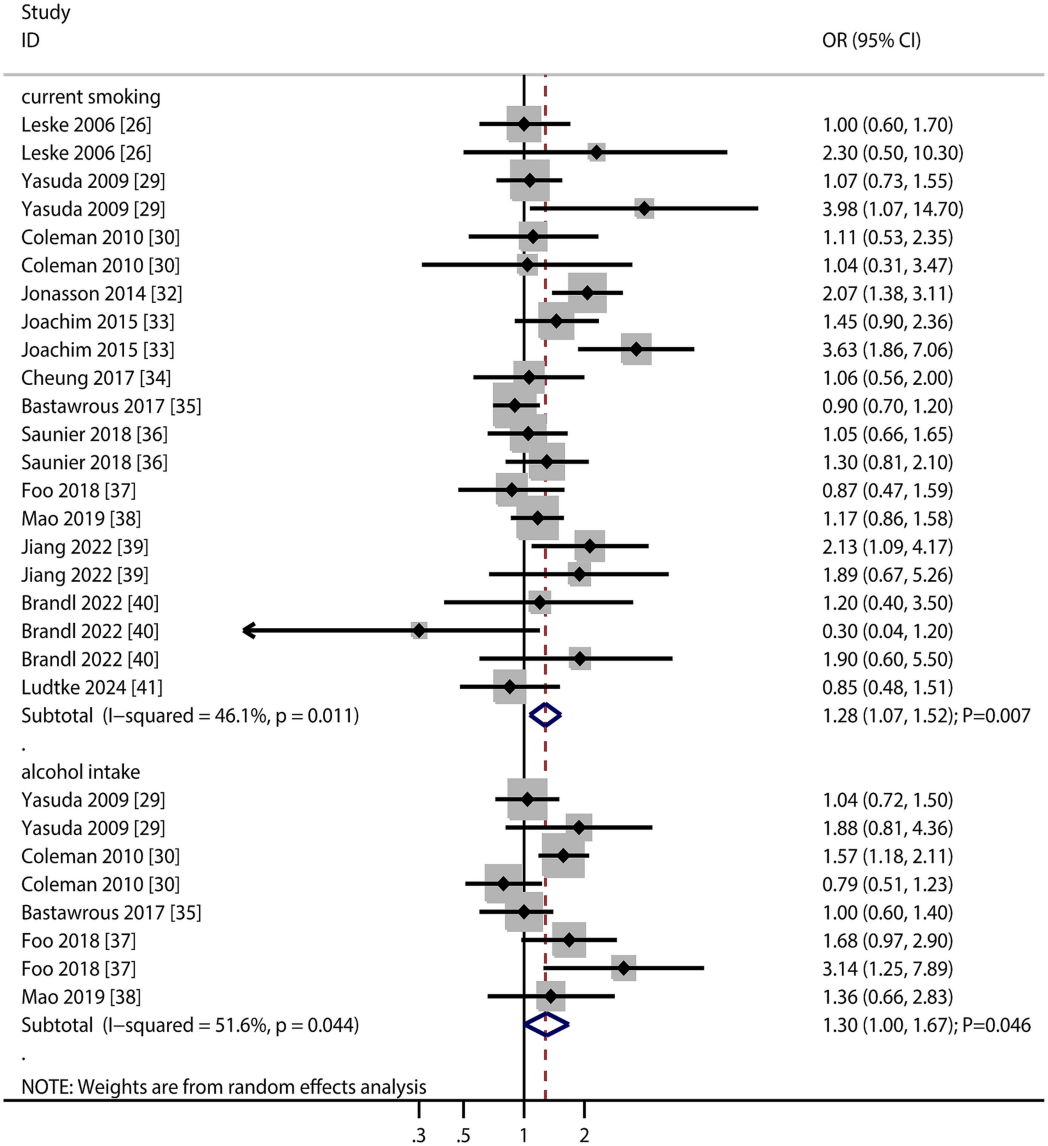
Association of smoking and alcohol intake with the risk of age-related macular degeneration (AMD).

The number of studies that reported the association of current smoking and alcohol with the risk of AMD was 13 and 5, respectively (Figure 3). We noted that current smoking (OR: 1.28; 95% CI: 1.07–1.52; *p* = 0.007) and alcohol intake (OR: 1.30; 95% CI: 1.00–1.67; *p* = 0.046) were associated with an increased risk of AMD. Moreover, significant heterogeneity was observed among the included studies in terms of current smoking status (*I*^2^ = 46.1%; *p* = 0.011) and alcohol intake (*I*^2^ = 51.6%; *p* = 0.044). The sensitivity analysis revealed that the pooled conclusion regarding the association between current smoking status and AMD was stable, whereas the association between alcohol intake and AMD was altered (Supplementary material 2). The subgroup analyses revealed that current smoking status was associated with an increased risk of late AMD, whereas alcohol consumption was associated with an increased risk of early AMD (Table 2). We did not find a significant publication bias regarding the association of current smoking (*p* value for Egger: 0.208; *p* value for Begg: 0.239) and alcohol intake (*p* value for Egger: 0.434; *p* value for Begg: 0.536) with the risk of AMD (Supplementary material 3).

The significant associations with current smoking and alcohol intake highlight modifiable lifestyle factors for intervention. Notably, smoking’s stronger association with late AMD suggests it accelerates disease progression, while alcohol may influence early-stage pathogenesis.

### Hypertension and diabetes mellitus

**Figure 4:**
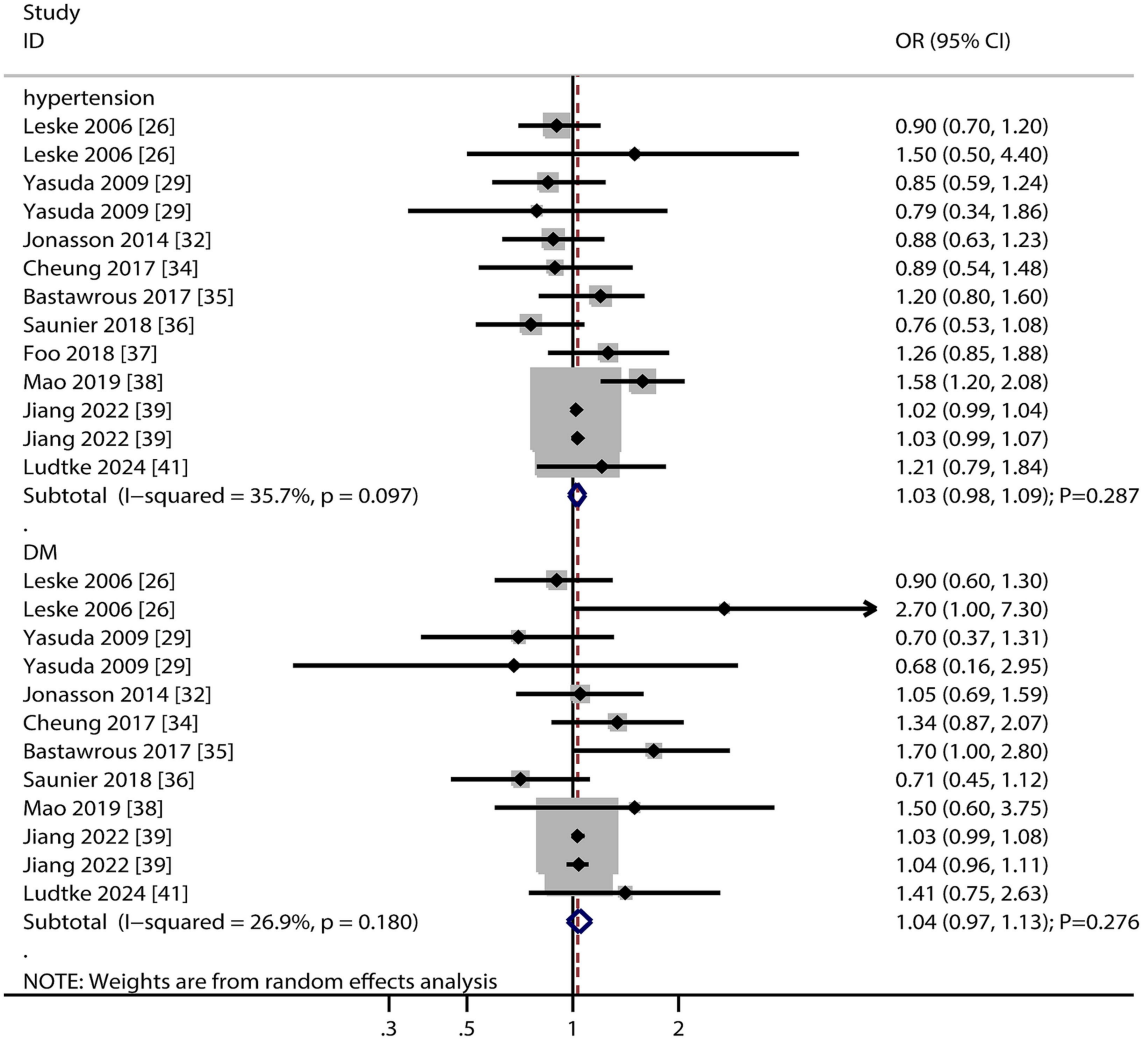
Association of hypertension and diabetes mellitus (DM) with the risk of age-related macular degeneration (AMD).

The number of studies reporting the association of hypertension and diabetes mellitus (DM) with the risk of AMD was 10 and nine, respectively (Figure 4). The summary results indicated that hypertension (OR: 1.03; 95% CI: 0.98–1.09; *p* = 0.287) and DM (OR: 1.04; 95% CI: 0.97–1.13; *p* = 0.276) were not associated with the risk of AMD, and potential significant heterogeneity for hypertension was observed (*I*^2^ = 35.7%; *p* = 0.097). The sensitivity analysis revealed that hypertension might be associated with an increased risk of AMD, whereas the relationship between DM and AMD was stable (Supplementary material 2). The subgroup analyses revealed that hypertension and DM were not associated with early or late AMD (Table 2). There were no significant publications regarding the association between hypertension (*p* value for Egger: 0.821; *p* value for Begg: 1.000) and DM (*p* value for Egger: 0.464; *p* value for Begg: 0.631) and the risk of AMD (Supplementary material 3).

The lack of significant associations between hypertension/DM and AMD risk possibly due to differences in patient demographics or comorbidity management. This finding warrants replication in more diverse cohorts.

### Lipid profiles

**Figure 5:**
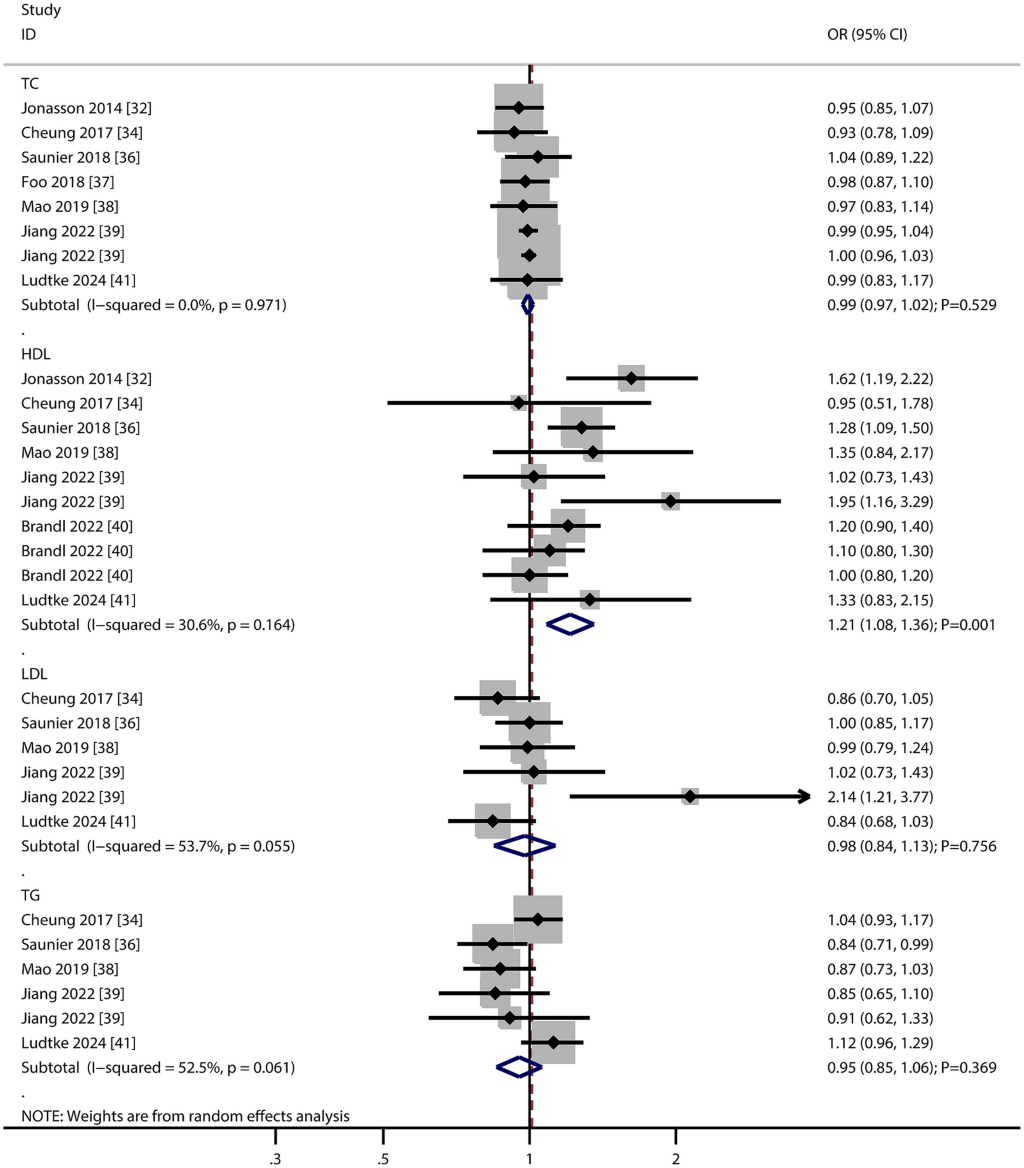
Association of total cholesterol (TC), high-density lipoprotein (HDL), low-density lipoprotein (LDL), and triglyceride (TG) levels with the risk of age-related macular degeneration (AMD).

The number of studies reporting the association of total cholesterol (TC), high-density lipoprotein (HDL), low-density lipoprotein (LDL), and triglyceride (TG) levels with the risk of AMD were seven, seven, five, and five, respectively (Figure 5). We noted that per 1 mmol/L increment in HDL was associated with an increased risk of AMD (OR: 1.21; 95% CI: 1.08–1.36; *p* = 0.001). In contrast, per 1 mmol/L increment in TC (OR: 0.99; 95% CI: 0.97–1.02; *p* = 0.529), LDL (OR: 0.98; 95% CI: 0.84–1.13; *p* = 0.756), and TG (OR: 0.95; 95% CI: 0.85–1.06; *p* = 0.369) were not associated with the risk of AMD. There was significant heterogeneity per 1 mmol/L increment in LDL (*I*^2^ = 53.7%; *p* = 0.055) and TG (*I*^2^ = 52.5%; *p* = 0.061) levels with the risk of AMD. The sensitivity analyses found a pooled conclusion regarding the association of each 1 mmol/L increment in TC, HDL, LDL, and TG with the risk of AMD was stable (Supplementary material 2). The subgroup analyses found that per 1 mmol/L increment in HDL was associated with an increased risk of early and late AMD. Moreover, per 1 mmol/L increment in LDL was associated with an increased risk of late AMD (Table 2). There were no significant publications regarding the association of TC (*p* value for Egger: 0.229; *p* value for Begg: 0.386), HDL (*p* value for Egger: 0.516; *p* value for Begg: 0.721), LDL (*p* value for Egger: 0.137; *p* value for Begg: 0.452), or TG (*p* value for Egger: 0.298; *p* value for Begg: 1.000) with the risk of AMD (Supplementary material 3).

The positive association with HDL is counterintuitive but aligns with recent studies suggesting oxidized HDL may promote retinal inflammation. The lack of association with other lipids highlights HDL as a potential biomarker for AMD risk stratification.

### Other factors

**Figure 6:**
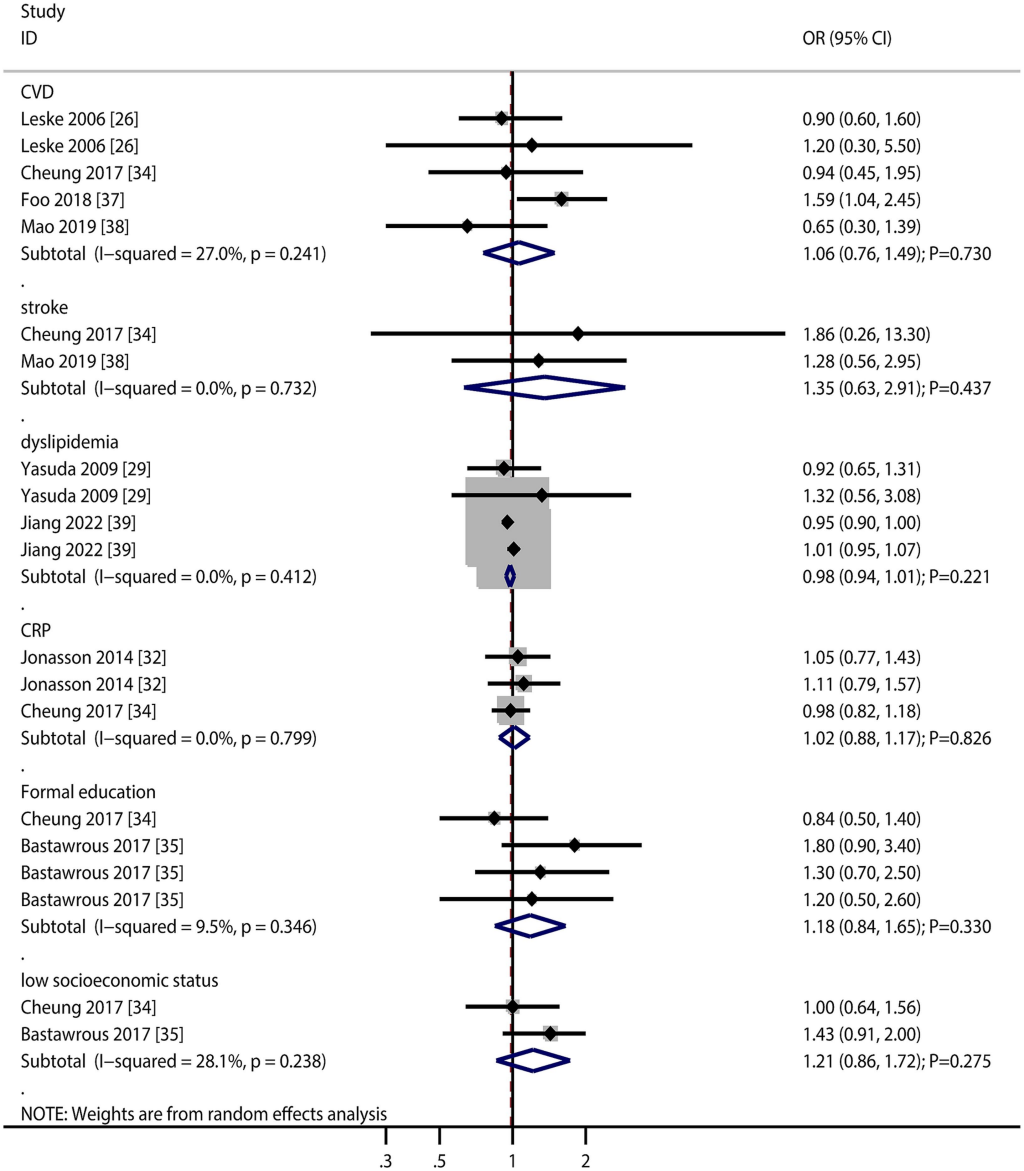
Association of cardiovascular disease (CVD), stroke, dyslipidemia, C-reactive protein (CRP), formal education, and low socioeconomic status with the risk of age-related macular degeneration (AMD).

The numbers of studies reporting the association of cardiovascular disease (CVD), stroke, dyslipidemia, C-reactive protein (CRP), formal education, and low socioeconomic status with the risk of AMD were four, two, two, two, two, and two, respectively (Figure 6). There were no significant associations regarding CVD (OR: 1.06; 95% CI: 0.76–1.49; *p* = 0.730), stroke (OR: 1.35; 95% CI: 0.63–2.91; *p* = 0.437), dyslipidemia (OR: 0.98; 95% CI: 0.94–1.01; *p* = 0.221), CRP (OR: 1.02; 95% CI: 0.88–1.17; *p* = 0.826), formal education (OR: 1.18; 95% CI: 0.84–1.65; *p* = 0.330), and low socioeconomic status (OR: 1.21; 95% CI: 0.86–1.72; *p* = 0.275) with the risk of AMD. Furthermore, we noted that total drusen >10% of grid (OR: 7.85; 95% CI: 4.66–13.23; *p* < 0.001), presence of depigmentation (OR: 6.39; 95% CI: 2.48–16.44; *p* < 0.001), presence of hyperpigmentation (OR: 6.03; 95% CI: 1.94–18.73; *p* = 0.002), and >10 small drusen (OR: 7.21; 95% CI: 2.10–24.72; *p* = 0.002) were associated with an increased risk of AMD, while refractive error was not associated with the risk of AMD (OR: 1.25; 95% CI: 1.00–1.58; *p* = 0.054).

**Figure 7:**
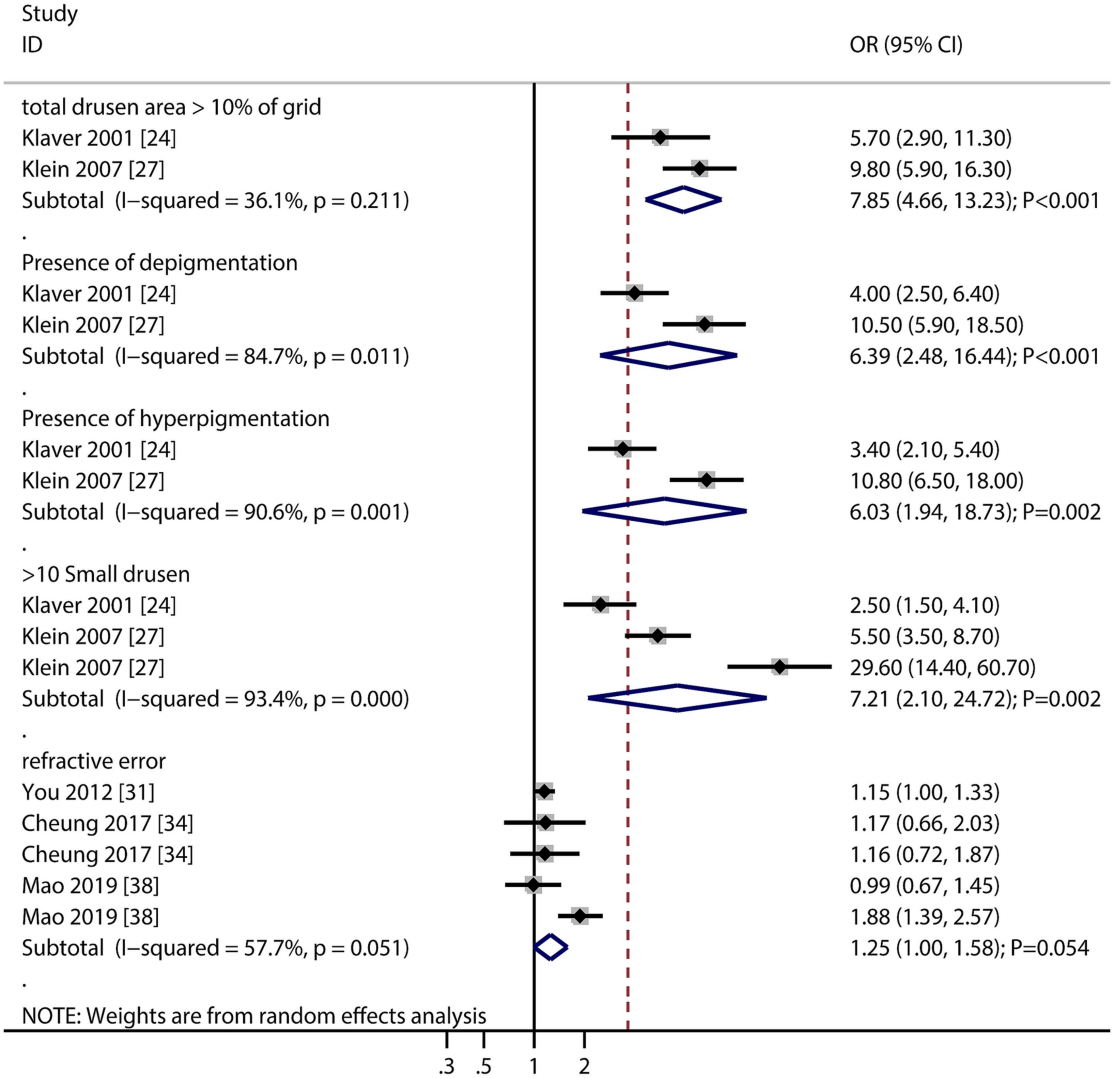
Association of total drusen >10% of the grid, presence of depigmentation, presence of hyperpigmentation, and >10 small drusens, and refractive error with the risk of age-related macular degeneration (AMD).

Retinal features (drusen burden, pigmentation changes) emerged as the strongest risk factors, underscoring their clinical utility in predicting AMD progression. The lack of association with refractive error contrasts with some studies, possibly due to methodological differences in refraction assessment (Figure 7).

## Discussion

The current cohort study aimed to investigate risk factors for the occurrence and development of AMD. This large-scale quantitative study included 38,697 participants from 18 cohort studies covering a wide population range. The results showed significant associations between an increased risk of AMD and the following factors: female sex, older age, smoking, alcohol consumption, higher HDL levels, total drusen area >10% of the grid, presence of depigmentation, and presence of >10 small drusen. In addition, we conducted sensitivity analyses to assess the stability of our findings and performed subgroup analyses to identify the risk factors for early and late AMD. The results showed that the risk factors for early AMD included older age, alcohol consumption, and higher HDL levels. In contrast, the risk factors for late AMD included older age, smoking, and higher HDL and LDL levels.

Our study found that the risk of AMD was higher in females than in males, and that differences in hormone levels could be a significant factor. Fluctuations in estrogen levels in females may affect vascular health and inflammatory responses, thereby increasing the risk of AMD (43). Additionally, the risk of AMD increased significantly with age, consistent with previous research (13). We also discovered that smoking can significantly increase the risk of developing AMD as it elevates oxidative stress levels and generates large amounts of free radicals, which damage retinal cells and blood vessels, accelerating the degeneration of the macula (44). Smoking also triggers chronic inflammatory responses, promoting the release of inflammatory mediators that further damage retinal tissue (45). Furthermore, alcohol consumption significantly increases the risk of AMD. Long-term alcohol consumption can lead to nutritional deficiencies, particularly in essential nutrients for eye health, such as vitamins A, C, and E, and zinc. These nutrients are crucial in protecting the retina from oxidative damage and maintaining normal function (46, 47). Finally, alcohol consumption can also cause vascular dysfunction, affecting the blood supply to the retina and exacerbating ischemia and malnutrition in the macula (48, 49).

Higher HDL cholesterol levels significantly increase the risk of AMD. The function of HDL depends on its quantity and quality. If HDL particles are dysfunctional or oxidized, they may lose their anti-inflammatory and antioxidant properties and instead promote inflammatory responses and oxidative stress, which can damage retinal cells. High HDL levels may be associated with abnormalities in lipid metabolism, leading to lipid deposition in the retinal blood vessels, which causes vascular damage and inflammation (50–52). Moreover, we found that a total drusen area >10% of the grid, depigmentation, and the presence of >10 small drusens can significantly increase the risk of AMD. These factors may increase the risk of AMD through the following mechanisms. First, a larger drusen area indicates a higher amount of lipid and protein deposition under the retina, which can lead to retinal cell dysfunction and inflammatory responses (53). Second, depigmentation may result from the damage or death of retinal pigment epithelial cells, further weakening the protective barrier of the retina and increasing oxidative stress and inflammation (54). Finally, the presence of numerous small drusen may reflect early pathological changes; these small deposits can gradually accumulate and coalesce over time, forming larger drusen and accelerating the degenerative process in the macula (55).

The subgroup analysis found certain differences in the risk factors between early and late AMD. Specifically, alcohol consumption increases the risk of early AMD, whereas smoking and high LDL levels increase the risk of late AMD. Alcohol consumption may elevate oxidative stress levels and generate free radicals, leading to damage to the retinal cells and blood vessels in the early stages, thereby initiating early lesions. In contrast, smoking and high LDL levels may cause severe vascular damage and retinal dysfunction through long-term chronic inflammatory responses and atherosclerotic processes, thereby promoting the development of late AMD.

This study had some limitations. First, it included both prospective and retrospective cohort studies, which may result in recall bias and uncontrolled confounding biases. Second, the diagnostic criteria and severity of AMD varied among the included studies, which may have significantly affected the effect sizes of risk factors. Third, the adjustment factors used in the included studies were inconsistent, which may have affected the stability of the results. Fourth, this study did not address hereditary factors (e.g., CFH and ARMS2 gene variants), as existing evidence primarily stems from cross-sectional or case–control studies, which fall outside our cohort study inclusion criteria. Future meta-analyses integrating genetic epidemiology may complement these findings. Finally, this study is based on published research, and the extracted data are aggregated, which limits further exploratory analyses to some extent.

Several unaddressed risk factors and mechanistic gaps merit investigation. First, genetic variants interact with environmental factors in AMD progression, but few cohort studies have explored gene–environment interactions. Genome-wide association studies combined with longitudinal data could clarify these interactions. Second, emerging biomarkers such as oxidized phospholipids and inflammatory cytokines (e.g., IL-6, TNF-α) show promise in predicting AMD progression but require validation in large cohorts. Third, most included studies were conducted in Caucasian populations, leaving a gap in understanding risk factor profiles in Asian, African, and Hispanic populations. Meta-regression by ethnicity may reveal regional disparities in drusen biology or lipid metabolism. Fourth, the role of dietary antioxidants and omega-3 fatty acids remains unclear due to reliance on case–control designs. Prospective cohort studies with standardized dietary assessment are needed. Finally, interventional trials testing whether modifying HDL levels or alcohol consumption slows AMD progression would bridge the translational gap between observational findings and clinical practice.

## Conclusion

Through meta-analysis of 18 cohort studies, we identified sex (female), age, smoking, alcohol consumption, elevated HDL, and specific retinal lesions (drusen area >10% of grid, depigmentation, and >10 small drusens) as robust risk factors for AMD progression. Notably, age and HDL influenced both early and late AMD, whereas smoking specifically exacerbated late-stage progression, and alcohol intake was associated with early AMD. These findings provide important insights into the mechanisms underlying AMD development and highlight the role of multiple risk factors at different stages.

## Data Availability

The original contributions presented in the study are included in the article/Supplementary material, further inquiries can be directed to the corresponding author.
