# Supplementary material for: Identification of risk factors for the progression of age-related macular degeneration: a systematic review and meta-analysis of cohort studies
Source: Front Med (Lausanne). 2025 Jul 23;12:1544765. doi: 10.3389/fmed.2025.1544765 (PMC12325228; doi:10.3389/fmed.2025.1544765)
Supplement: Supplementary file 1 [file Data_Sheet_1.docx]

**Search strategy in PubMed:**

{“Macular Degeneration” [Mesh] AND (“Epidemiology” [Mesh] OR “Longitudinal Studies” [Mesh] OR “Cohort Studies” [Mesh])}; {(“age-related maculopathy” [All Fields] OR “age-related maculopathy” [All Fields] OR “age-related macular degeneration” [All Fields] OR “age-related macular degeneration” [All Fields] OR “macular degeneration” [All Fields]) AND (“progression” [All Fields] OR “epidemiology” [All Fields] OR “risk factors” [All Fields])}
