# Supplementary material for: Identification of risk factors for the progression of age-related macular degeneration: a systematic review and meta-analysis of cohort studies
Source: Front Med (Lausanne). 2025 Jul 23;12:1544765. doi: 10.3389/fmed.2025.1544765 (PMC12325228; doi:10.3389/fmed.2025.1544765)
Supplement: Supplementary file 3 [file Data_Sheet_3.docx]

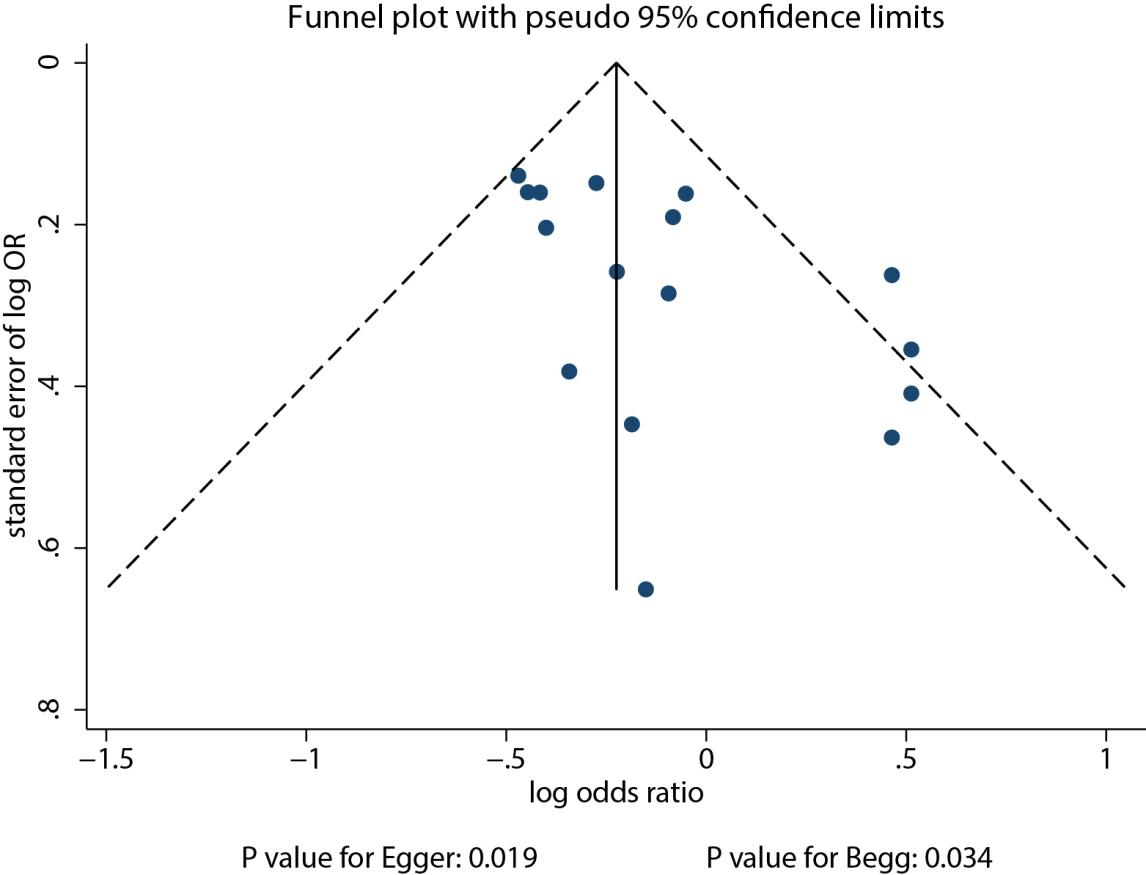


Figure S1. Funnel plot for male vs female on the risk of AMD


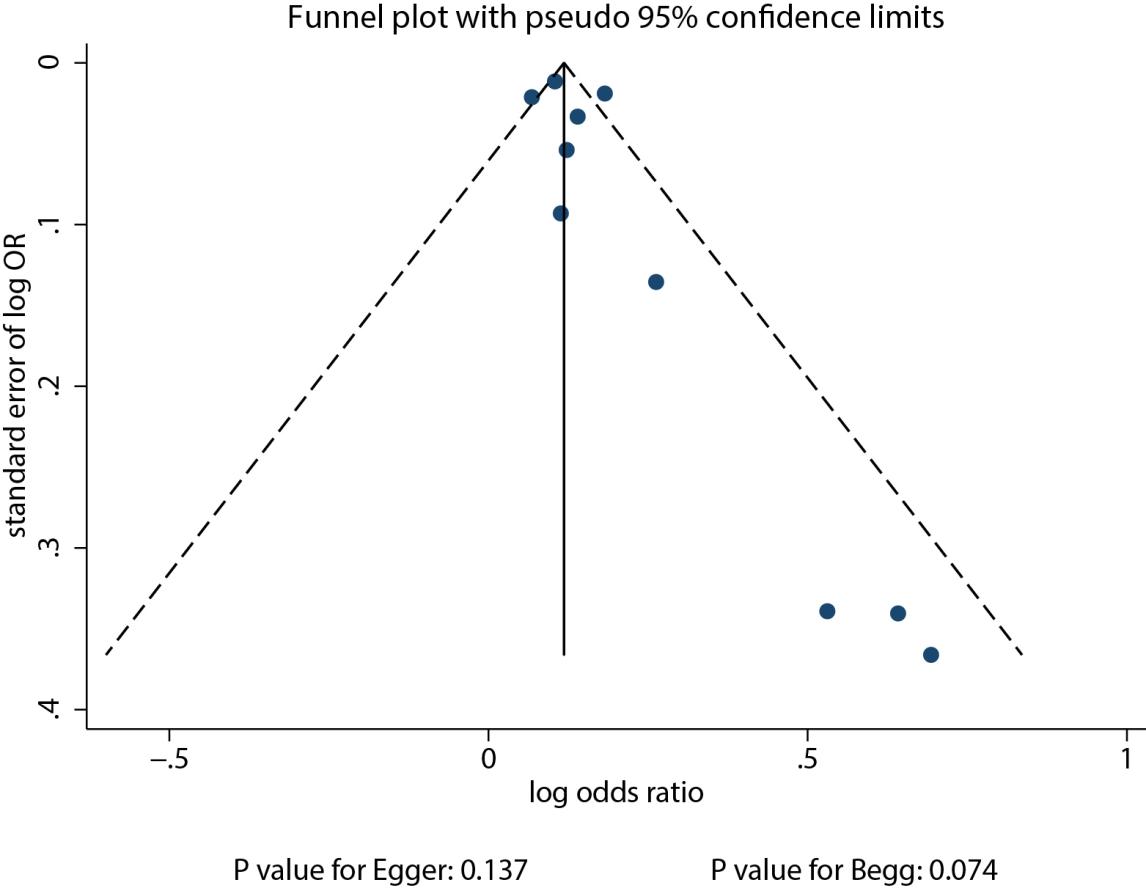


Figure S2. Funnel plot for the association of per 5 year increment in age with the risk of AMD


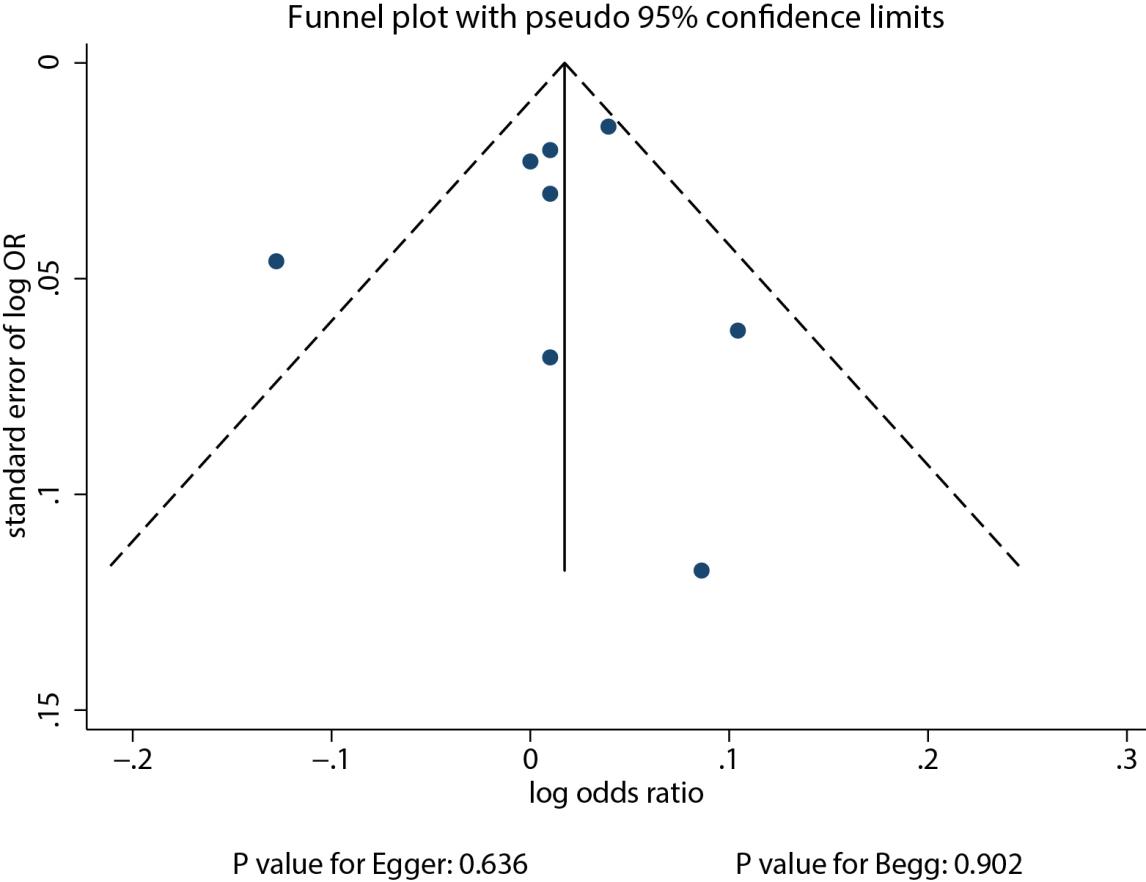


Figure S3. Funnel plot for the association of per 1 kg/m^2^ increment in BMI with the risk of AMD


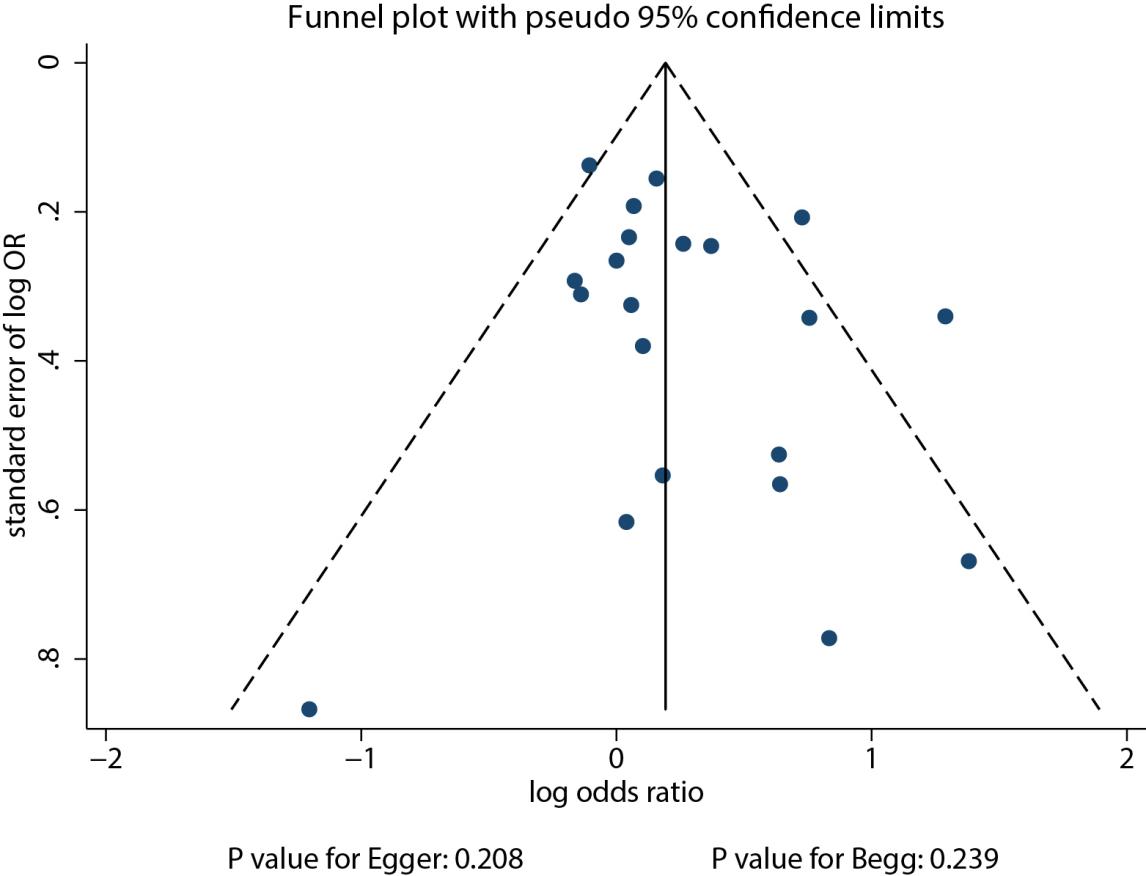


Figure S4. Funnel plot for the association of current smoking with the risk of AMD


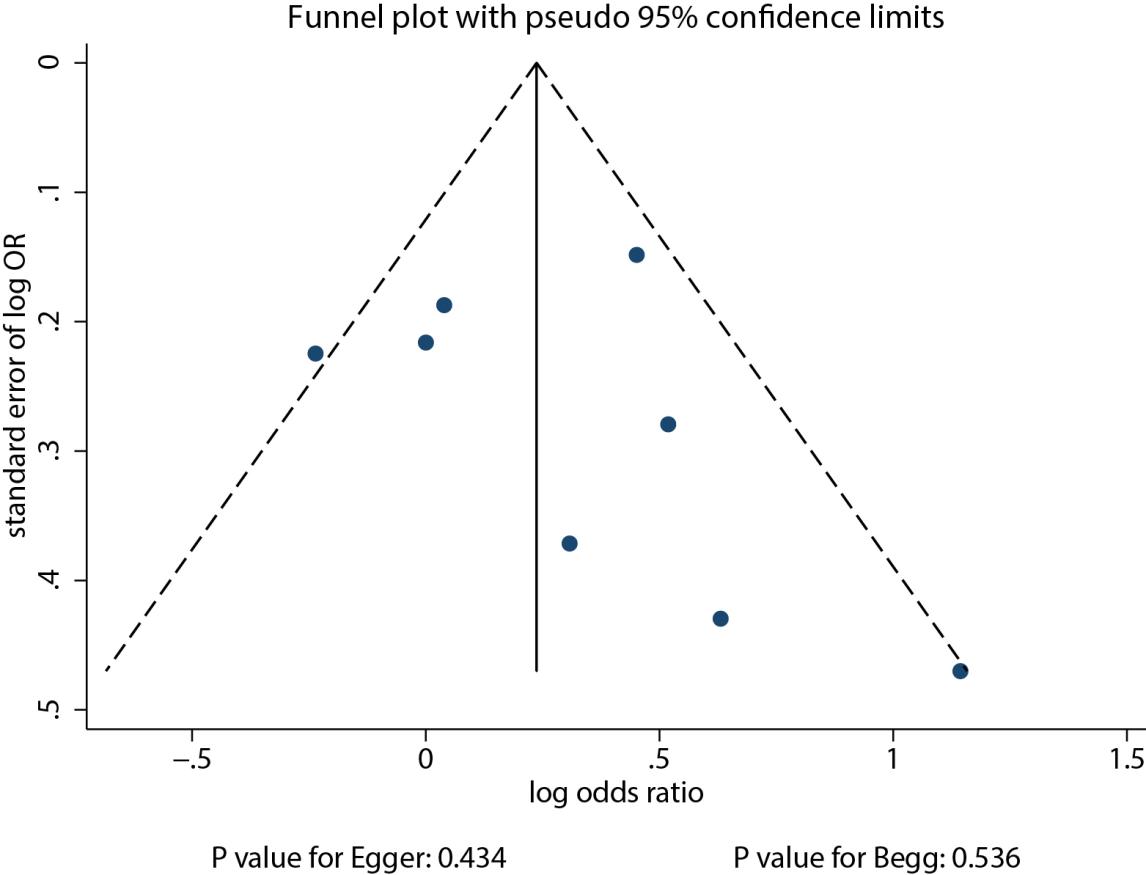


Figure S5. Funnel plot for the association of alcohol intake with the risk of AMD


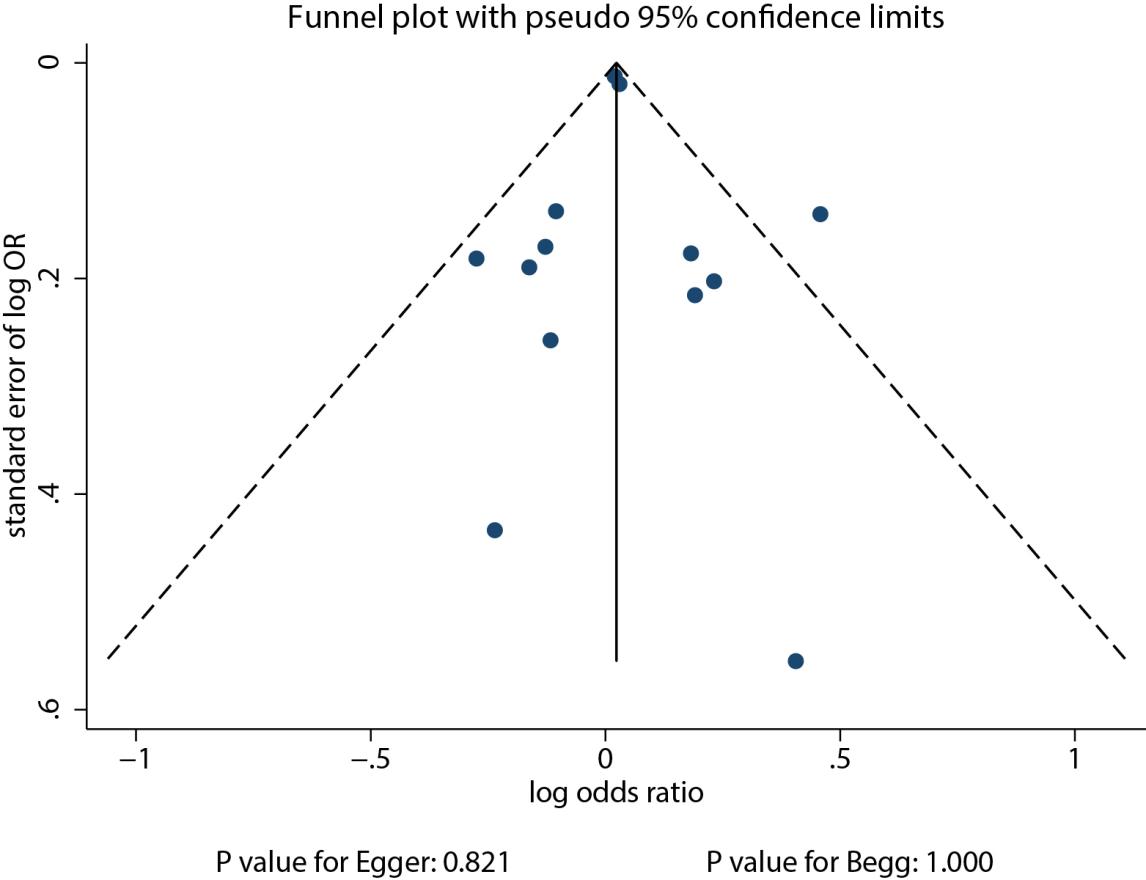


Figure S6. Funnel plot for the association of hypertension with the risk of AMD


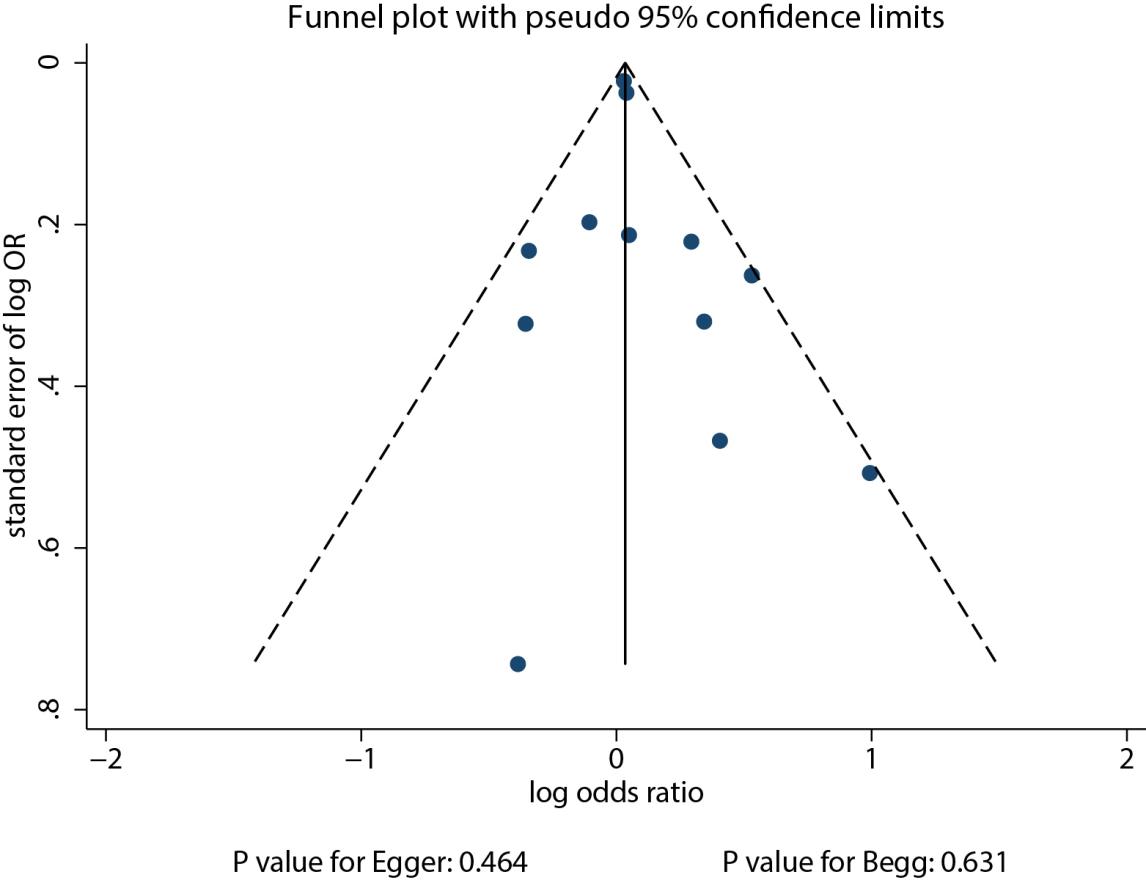


Figure S7. Funnel plot for the association of DM with the risk of AMD


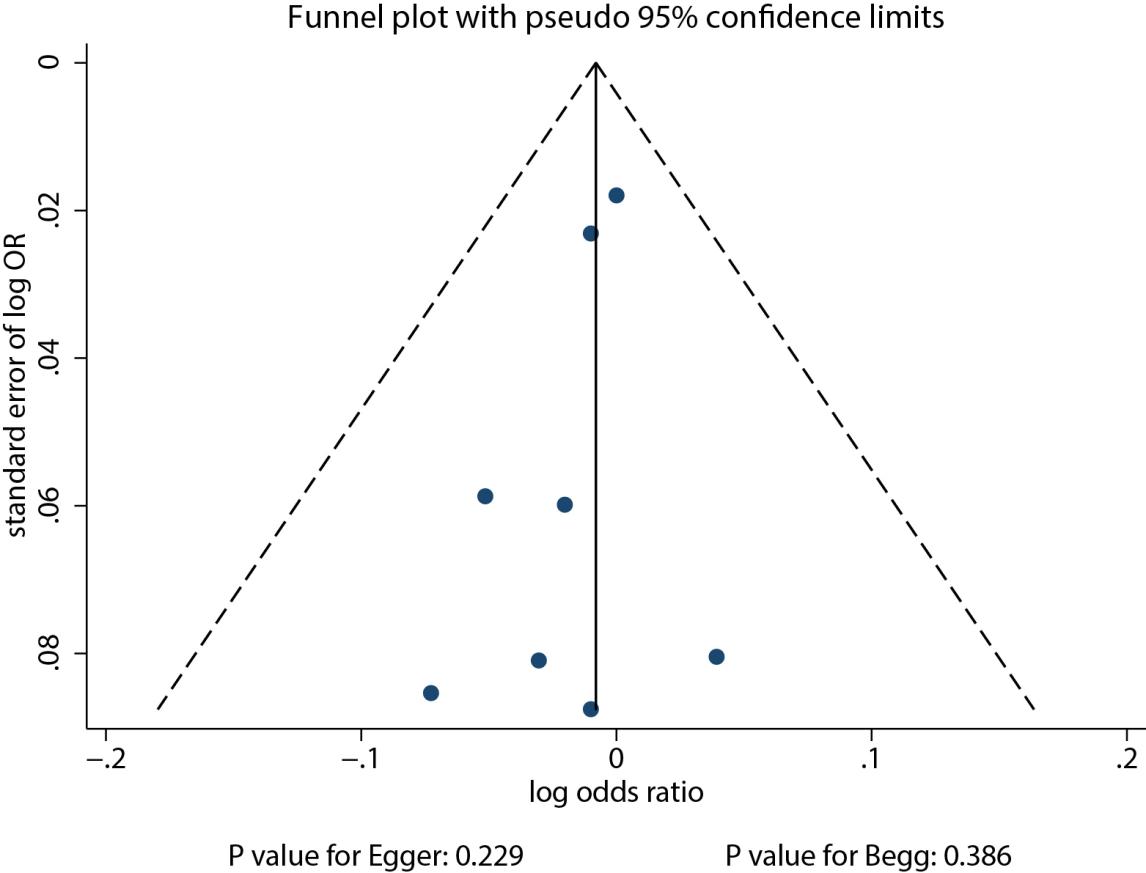


Figure S8. Funnel plot for the association of per 1 mmol/L increment in TC with the risk of AMD


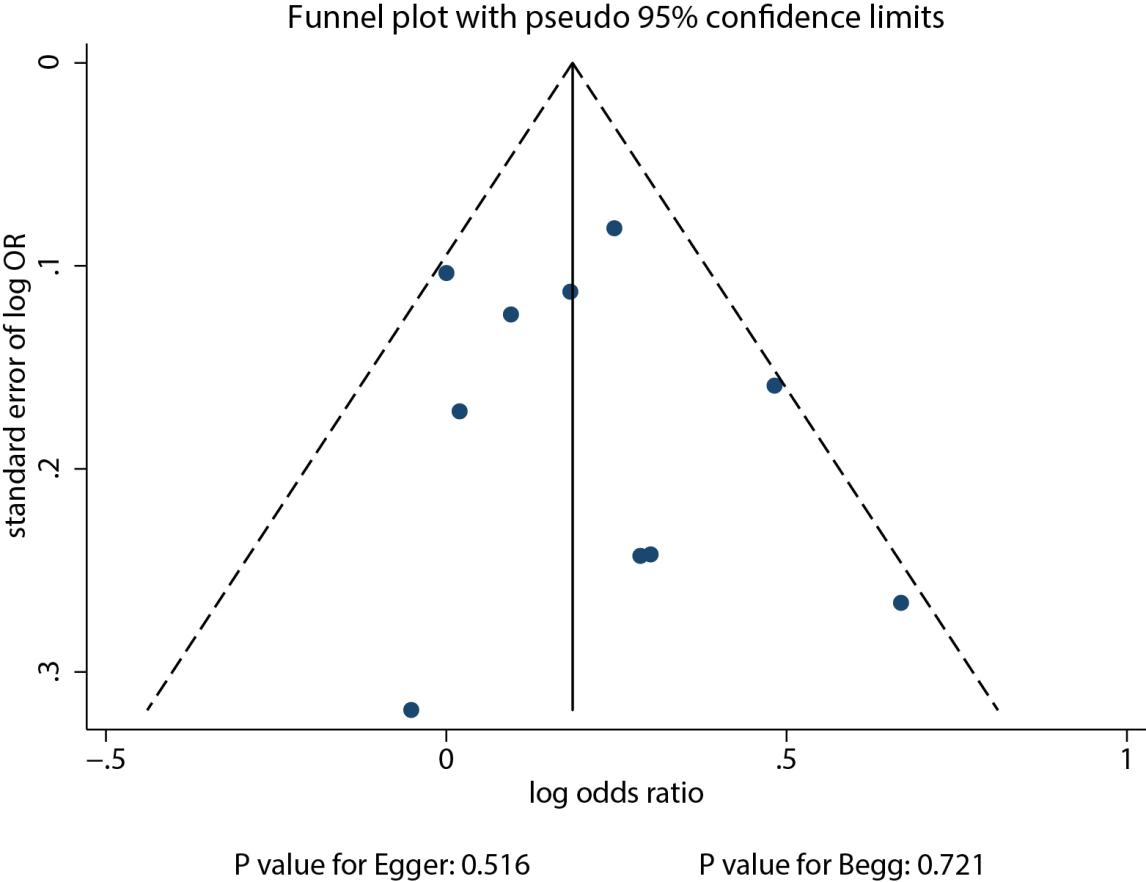


Figure S9. Funnel plot for the association of per 1 mmol/L increment in HDL with the risk of AMD


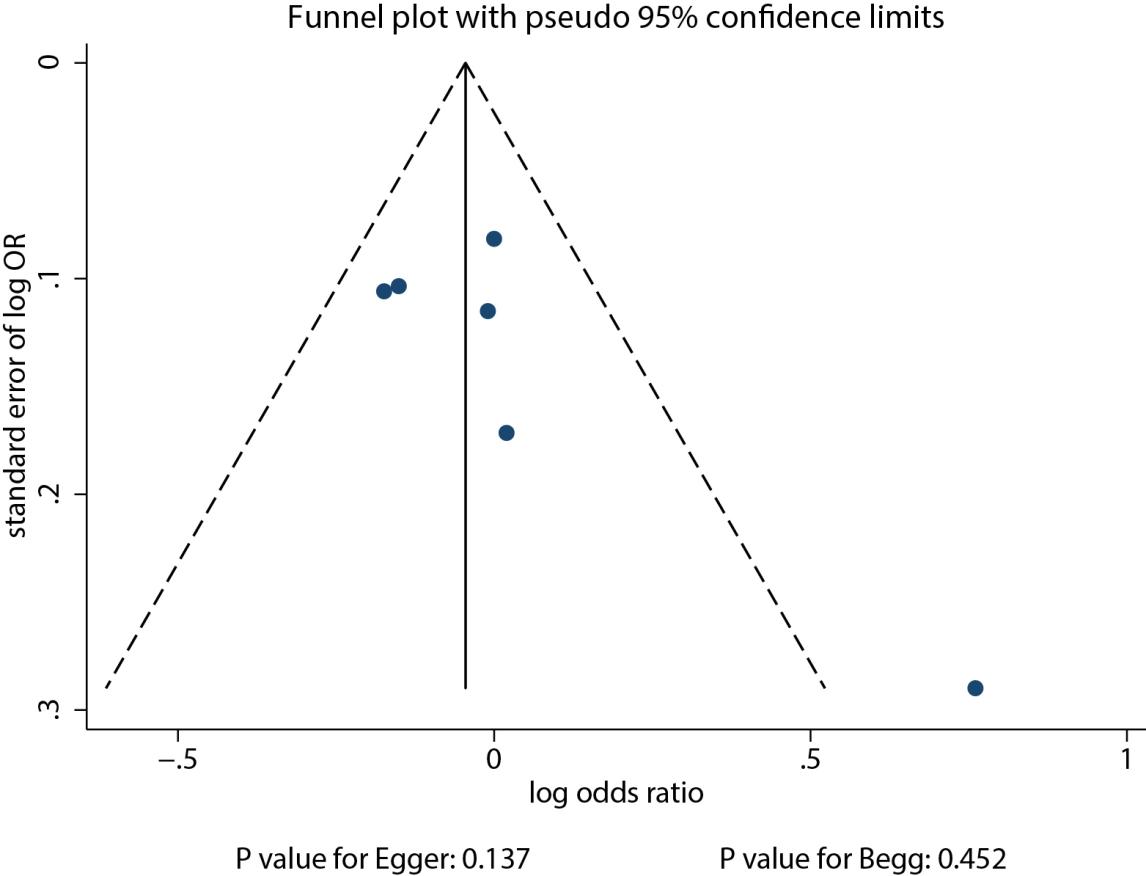


Figure S10. Funnel plot for the association of per 1 mmol/L increment in LDL with the risk of AMD


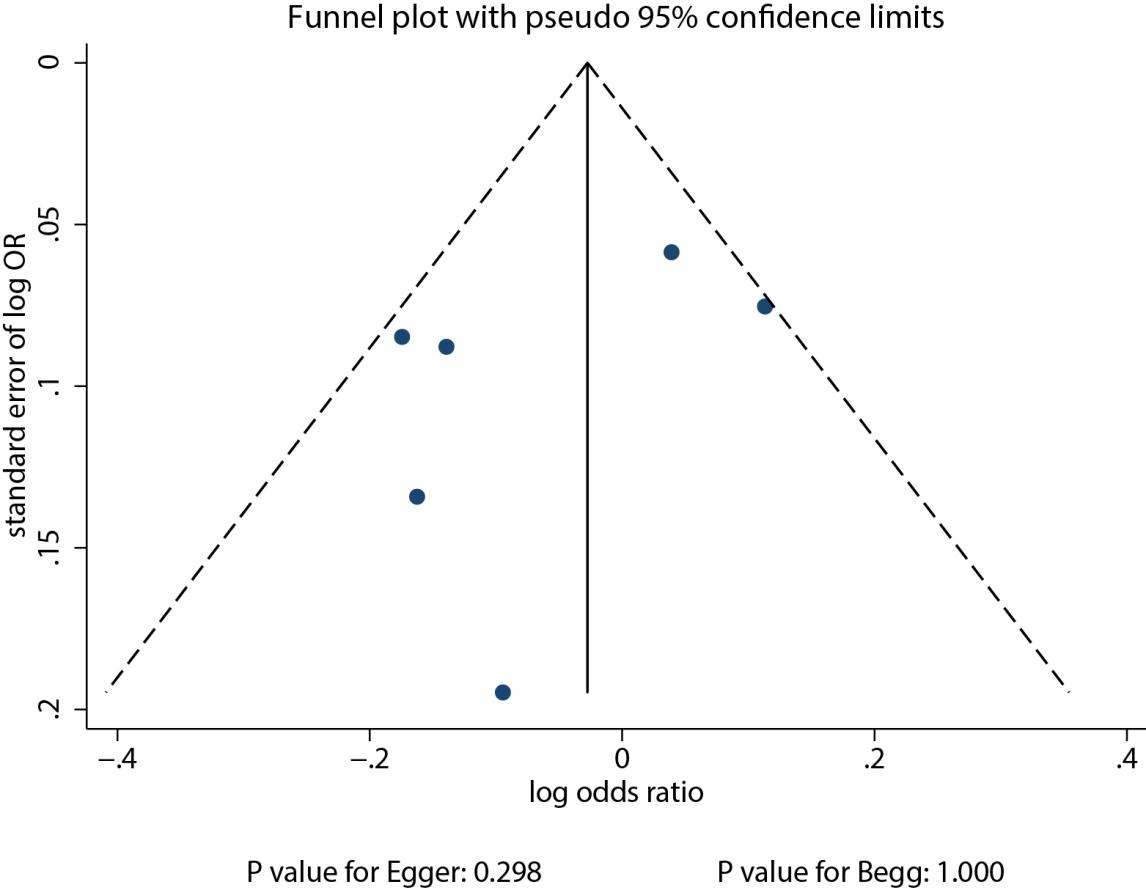


Figure S11. Funnel plot for the association of per 1 mmol/L increment in TG with the risk of AMD
